# Stabilising selection on immune response in male black grouse *Lyrurus tetrix*

**DOI:** 10.1007/s00442-017-4014-1

**Published:** 2017-11-24

**Authors:** Carl D. Soulsbury, Heli Siitari, Christophe Lebigre

**Affiliations:** 1 School of Life Sciences, University of Lincoln, Brayford Pool, Lincoln, UK; 2 Department of Biological and Environmental Sciences, University of Jyväskylä, P.O. Box 35, 40014 Jyväskylä, Finland; 3 IFREMER, Centre Brest, 29820 Plouzané, France

**Keywords:** Ecological immunology, ELISA, Immunocompetance, Life history theory, Stabilising selection

## Abstract

Illnesses caused by a variety of micro- and macro- organisms can negatively affect individuals’ fitness, leading to the expectation that immunity is under positive selection. However, immune responses are costly and individuals must trade-off their immune response with other fitness components (e.g. survival or reproductive success) meaning that individuals with intermediate response may have the greatest overall fitness. Such a process might be particularly acute in species with strong sexual selection because the condition-dependence of male secondary sexual-traits might lead to striking phenotypic differences amongst males of different immune response levels. We tested whether there is selection on immune response by survival and reproduction in yearling and adult male black grouse (*Lyrurus tetrix*) following an immune challenge with a novel antigen and tested the hypothesis that sexual signals and body mass are honest signals of the immune response. We show that yearling males with highest immune response to these challenges had higher survival, but the reverse was true for adults. Adults with higher responses had highest mass loss and adult males with intermediate immune response had highest mating success. Tail length was related to baseline response in adults and more weakly in yearlings. Our findings reveal the complex fitness consequences of mounting an immune response across age classes. Such major differences in the direction and magnitude of selection in multiple fitness components is an alternative route underpinning the stabilising selection of immune responses with an intermediate immune response being optimal.

## Introduction

Within natural populations, individuals are constantly exposed to parasitic organisms that can have significant detrimental effects on their fitness (Poulin 2007). Infection and damage by parasites may be resisted by mounting an immune response, and so immune responses should be under positive directional selection (Råberg et al. 2000). However, mounting an immune response is complex as there are many potential trade-offs within the immune system (e.g. humoral vs cellular response, innate vs acquired immune responses; Norris and Evans 2000) and immune responses are costly (Nordling et al. 1998; Råberg et al. 2000; Råberg and Stjernman 2003; Møller and Saino 2004; Van der Most et al. 2011). Such costs may either lead to a degree of tolerance against pathogens and parasites or lead to trade off with other life-history traits (Sheldon and Verhulst 1996; Harshman and Zera 2007) such as survival (Moret and Schmid-Hempel 2000; Hanssen et al. 2004; Jacot et al. 2004; Møller and Saino 2004; Eraud et al. 2009) or reproductive success (Uller et al. 2006). A high immune response may, therefore, lower individuals’ fitness through increased energetic costs (Svensson et al. 1998; Martin et al. 2003; Eraud et al. 2005), or physiological costs due to the damage caused by the activated immune system, e.g. non-specific immune responses such as fever and cytotoxins (Sadd and Siva-Jothy 2006; Sorci and Faivre 2009). Conversely, a too-low immune response may lead to lower fitness because of higher parasite burdens (Hayward et al. 2011). Consequently, the immune response is expected to be under stabilising selection with an intermediate response that manages or balances resistance versus tolerance, but does not necessarily eliminate, infection being optimal (Viney et al. 2005; Stjernman et al. 2008; Råberg et al. 2009; Graham et al. 2010).

In many species, a key fitness component is the ability of males to attract mates. Secondary sexual traits may have evolved in part to signal resistance to parasites (Hamilton and Zuk 1982), and there is considerable evidence that sexual ornaments can honestly signal immune responses and immunocompetence (Saino et al. 1997; Zuk and Johnsen 1998; Mougeot et al. 2004; Loyau et al. 2005; Bonato et al. 2009). Females may, therefore, use signals as honest cues of male genetic quality relating to parasite resistance (Dunn et al. 2013). Hence in addition to survival (i.e. natural selection), mating success (i.e. sexual selection) may have a significant role in driving optimal immune response.

Currently there is some evidence to suggest that intermediate immune responses may represent the optima. In blue tits (*Cyanistes caeruleus*), survival was highest at both intermediate parasite loads and intermediate levels of the primary humoral immune responsiveness to diphtheria and directional selection in the secondary response to tetanus (Råberg and Stjernman 2003; Stjernman et al. 2008). In Soay sheep (*Ovis aries*), high antibody titres were associated with higher overwinter survival but reduced fecundity, meaning that individuals had similar overall fitness irrespective of their antibody levels (Graham et al. 2010) and to our knowledge, selection on immune response by multiple components of fitness have only been studied in Soay sheep (Graham et al. 2010).

In this study, we quantified the magnitude of natural and sexual selection acting on immune response in yearling and adult male black grouse (*Lyrurus tetrix*). Male black grouse are under strong sexual selection, with females using multiple condition-dependent ornaments (tail (lyre) length, blue structural colouration of feathers, red eye comb size) and behaviours (fighting, lek attendance, distance from lek centre) to select males (Alatalo et al. 1991; Hovi et al. 1994; Höglund et al. 1997; Rintamäki et al. 2001; Hämäläinen et al. 2012; Kervinen et al. 2016). Lekking is energetically demanding (Vehrencamp et al. 1989), with males losing considerable body mass during the breeding season (Lebigre et al. 2013) through their investment in lekking activity (Nieminen et al. 2016). Hence, it might be predicted that the energetic demands of a too-high immune response are costly for males, whereas a too-low response may make males vulnerable to parasites, also diminishing male performance. At the same time, different life stages may invest differently in immune response; juvenile males do not invest heavily in costly mating effort (Kervinen et al. 2012) or in large sexual ornaments (Kervinen et al. 2015), so may not face a trade-off between immune function and mating effort. Thus, selection acting either on male survival (viability selection) and mating success (sexual selection) may operate differently at different ages. We tested the combined role of viability and sexual selection on adult and yearling male grouse immune response using a diphtheria-tetanus vaccine with or without the anthelmintic Levamisole hydrochloride. We first measured the humoral response of male black grouse to tetanus-diphtheria and tested whether (a) sexual ornaments and body mass predicted the initial antibody level or the peak response, and then whether initial antibody level or peak response were related to (b) male overwinter survival, (c) attendance at the lek and (d) male mating success.

## Materials and methods

### Study site and capture methods

**Fig. 1:**
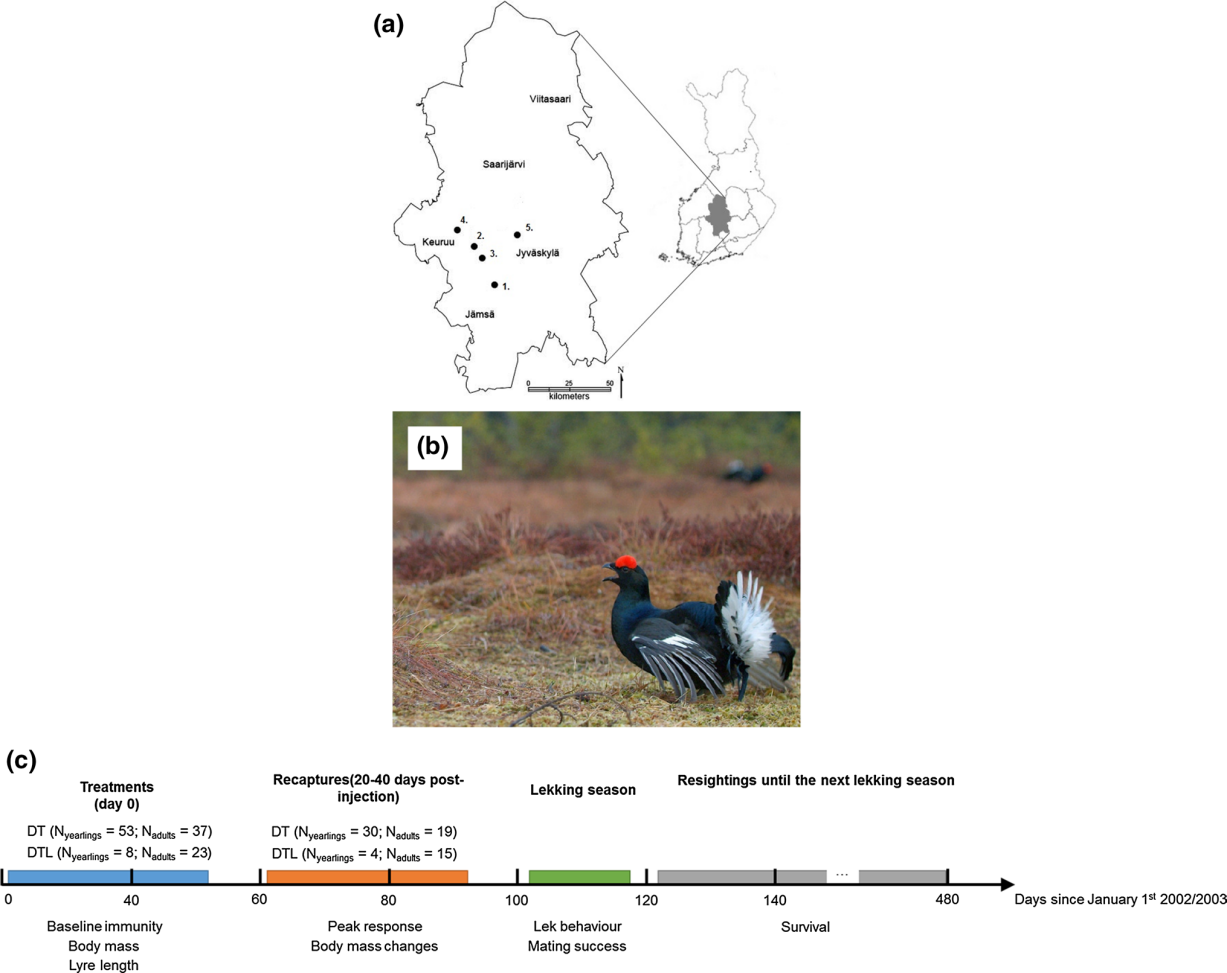
**a** Location of study sites (filled circles) in central Finland. 1: Köskenpaa, 2: Lehtosuo, 3: Teerisuo 4: Kummunsuo. 5: Valkeisuo; **b** an image of a male black grouse on the lek (photo by Gilbert Ludwig) and **c** timeline of the experiment carried out over 2002 and 2003. DT: Diphtheria-tetanus vaccination; DTL: Diphtheria-tetanus vaccination and levamisole treatment

Experimental vaccinations were carried out during the winters of 2002 and 2003, using male black grouse captured at five different winter flocks in Central Finland (Teerisuo, Valkeisuo, Lehtosuo, Kummunsuo, Koskenpaa; Fig. 1a, b). Birds were captured using walk-in traps baited with oat seeds. After capturing, males were weighed (to the nearest 10 g) with Pesola spring balance, and the maximum length of the lyre was measured (the length of the longest outer tail feathers). Males were aged as yearlings or older (based on the colour of their wing; Helminen 1963) and ringed with aluminium rings as well as marked with an individual colour ring combinations for the observations on the lek. A blood sample (1–2 ml) was taken from the brachial vein of all males, centrifuged at 16,000 rpm and plasma was stored at − 80 °C until analysis. As well as vaccination of all captured 2002 and 2003 males, a subset of birds (initial capture: adults = 23, yearlings = 8, peak response recaptures: adults = 15, yearlings = 4) were dosed with levamisole hydrochloride to remove underlying parasite loads. As we do not know the existing parasite load of each bird, levamisole treatment of some individuals allows us to look at the effect of vaccination on its own and in conjunction with existing parasite load.

### Experimental immune challenge and medication

A full timeline of the experiment is given in Fig. 1c. Experimental males were injected with 300 µl of diphtheria-tetanus vaccine in the pectoral muscle (Finnish National Public Health Institute, diphtheria 38 Lf -limit of flocculation- and tetanus 10 Lf, mixed with adjuvant aluminium phosphate at 1.0 mg/ml). Diphtheria-tetanus is a commonly used to induce artificial immune responses in birds (Hasselquist and Nilsson 2012).

To measure the antibody response against tetanus, males were recaptured repeatedly and blood sampled throughout the winter. These repeated measurements enabled us to measure both the baseline and peak immune responses. Reproductive success in male black grouse is condition-dependent and reproductive skew is high on leks (Alatalo et al. 1992; Kervinen et al. 2016). Leks act as units of competing males and any sham-injected males would be predicted to have higher fitness if mounting an immune response is costly. However, an accurate quantification of such an effect in black grouse using control and treatment groups would be extremely challenging because only a subset of the males captured within winter flocks display on the studied leks and a handful of them manage to mate. Hence, we chose to focus on parameters linked to the dynamics of the immune response of the birds (baseline and peak levels) rather than a direct comparison of vaccinated and sham-injected birds and acknowledge this limitation of our study.

Specific antibodies against tetanus were measured using ELISA with commercial anti-chicken antibodies (Sigma© 6409); samples were run in duplicate. We also quantified diphtheria antibodies, but samples run in duplicate showed no repeatability suggesting that the assay was unreliable. Anti-chicken immunoglobulin G (IgG) Ab can be used to determine antigen-specific and total Ig concentrations in plasma of wild birds (Müller et al. 2004). ELISA-plates (Cliniplate EB, Thermolabsystems, Helsinki, Finland) were first coated with an antigen (tetanus toxoid, National Public Health Institute, Helsinki, Finland). Samples and standards were added into the wells and incubated for 3 h at room temperature. After washing the plates, alkaline phosphatase conjugated anti-chicken IgG antibody (A-9171, Sigma Chemical Co. St Louis, Mo, USA) was added to the wells and incubated overnight at 4 °C. Finally, alkaline phosphatase substrate pNPP (p-nitrophenyl phosphate, Sigma 104^®^ phosphatase substrate, Sigma Chemical Co. St Louis, Mo, U.S.A) in 1 M diethanol amine buffer (1 mg ml^−1^) was applied. The absorbance of the plates was read in an ELISA reader at 405 nm. The samples, standards and conjugated antibodies were diluted in 1% BSA (bovine serum albumin, Fraction V, Roche Diagnostics GmbH, Mannheim, Germany) prepared in PBS. 1% BSA-PBS was also used for masking the wells before applying the samples. After each incubation step, the plates were washed three times with PBS-0.05% Tween 20. The assay was calibrated with a series of diluted standard samples applied on every plate. As a standard, pooled plasma from all immunised individuals measured was used. An arbitrary concentration of 10^6^ Units ml^−1^ (U ml^−1^) was given to the standard, and concentration of samples in each assay was expressed as U ml^−1^.

### Annual mating success and survival assessment

We monitored the lekking behaviour and mating success of male black grouse from late April to early May (i.e. during the mating season). These observations were undertaken daily (0300–0900 hours) from hides located in the vicinity of the leks,. We drew activity maps at regular intervals and recorded the spatial location and current behaviour of each male (inactive, hissing, rookooing, or fighting; Höglund et al. 1997). Female presence on the leks was also recorded. All copulations were documented and partners identified (if ringed). We estimated each male’s lek attendance (proportional to the highest attending male on the same lek). Males that were recorded in ≥ 30% of the activity maps and in ≥ 50% of the observation days were classified as territorial and males that visited the leks less frequently than described above were classified as non-territorial and not attending the lek (Kervinen et al. 2012).

Males that were not captured or observed in the following year (all leks within 3 kilometres from the capturing place were also monitored) were regarded as dead. Males are very philopatric to their lekking sites (Lebigre et al. 2008), and lack of subsequent capture or sighting is a good proxy for survival. We cannot rule out the possibility that some males that were not observed may have dispersed to other unknown lekking areas, but male dispersal propensity is generally low and juvenile-biased (Alatalo et al. 1991; Caizergues and Ellison 2002; Warren and Baines 2002).

### Statistical analysis

Experimental immune responses typically show a quadratic relationship, so we considered two key parts of the response: baseline antibody titres at day 0 (hereafter baseline antibody level) and antibody titre levels during the peak response (days 20–40: hereafter peak response). The baseline antibody levels typically correlate with other natural antibodies, suggesting that they reflect the basic level of (polyclonal) natural antibodies in circulation, i.e. a baseline measure of immunocompetence (Mendes et al. 2006). The peak response was chosen based on the consistent pattern of males having their highest response during this period.

We first tested whether long-term measures of male quality (body mass at initial capture, lyre length) reflected baseline or peak response levels. We used linear models with tetanus level as the dependent variable and trait size as covariates. For peak response, levamisole treatment (yes/no) was included as a fixed factor. Models were run separately for yearlings and adults, as there is considerable age-dependency on sexual trait values (Kervinen et al. 2015).

We assessed whether the immune response was costly by subtracting initial body mass levels from those collected during the peak response. We then carried out a linear model with mass change as the dependent variable and peak response as a covariate, male age as a fixed factor and the interaction between male age and peak response.

We compared overwinter survival and baseline antibody level using a binomial generalised linear model with age (yearling, adult), log tetanus levels and their interaction as fixed factors. We then tested whether peak response values were related to overwinter survival (1 = alive, 0 = dead) using a binomial generalised linear model with levamisole treatment (levamisole/null) and log tetanus level as fixed factors. We then assessed whether the mass change caused by the immune response was linked to survival. We carried out a binomial GLM with survival as the dependent variable and mass change and levamisole treatment as fixed factors. We carried this out separately for adults and yearlings.

High attendance at the leks is important for male mating success (Rintamäki et al. 2001), so we tested whether males that attended the lek and held a territory (yes/no) was related to log tetanus antibody level and levamisole treatment. Then using territorial males only, we tested whether male survival was related to antibody level. Sample sizes of non-territorial males was too low to analyse (*N* = 11).

For male mating success, we carried out Poisson generalised linear mixed effects model (GLMM) with treatment and log tetanus level (either baseline or peak response level) as fixed factors and lekking site (year and site combined) as a random factor. We included both linear and quadratic terms in the model to test for selection toward the intermediate response level. All analysis was run in R 2.15.2 (R Development Core team 2012), with generalised linear-mixed effects models run using the lme4 package (Bates et al. 2011).

## Results

### Tetanus response

In total, we caught 121 birds with 68 recaptures and resampled during the peak response (Fig. 1c). Baseline antibody level on day 0 correlated with peak values for yearlings (Spearman’s rank order correlation: *r*_S_ = 0.35, *p* = 0.042), but not adults (Spearman’s rank order correlation: *r*_S_ = 0.08, *p* = 0.668). Adult males (12.80 ± 0.08) had higher baseline antibody levels than yearlings (12.52 ± 0.10; Welch’s 2 sample *t* test: *t*_112.11_ = − 2.21, *p* = 0.029) and higher peak responses (adults: 14.06 ± 0.20; yearlings: 13.35 ± 0.19; 2 sample *t* test: *t*_66_ = − 2.61, *p* = 0.011).

### Tail length and immune response

**Fig. 2:**
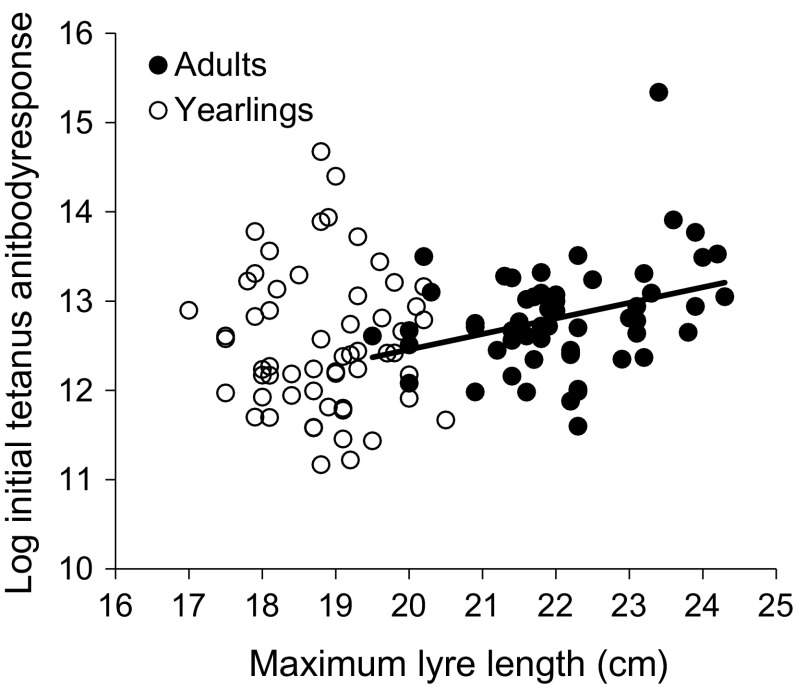
The relationship between (maximum lyre length (cm) and log tetanus antibody level at initial capture for adult males (solid points; *N* = 58) and yearling males (*N* = 58). The significant regression line for adult males is shown (*y* = 8.97 + 0.17*x*)

For yearlings, tail length only tended to be positively related to baseline antibody level (Table 1). By contrast, lyre length was significantly positively related to adult male baseline antibody level (Table 1; Fig. 2). For yearling and adult males, no phenotypic trait was significantly related to peak antibody response (Table 1), but in both cases the relationship with tail length tended to be positive in yearlings and adult males (Table 1).

**Table 1:** Results of linear regression models for phenotypic traits (maximum lyre length, body mass), treatment (with or without Levamisole) and initial or peak antibody levels for yearling and adult male black grouse

Age	Variable	Fixed factor	*β*	±SE	*t*	*P*
Yearling	Initial antibody level	Maximum lyre length (cm)	− 0.03	0.13	− 0.21	0.835
		Log body mass (g)	2.58	2.32	1.11	0.270
Yearling	Peak response	Maximum lyre length (cm)	0.50	0.28	1.78	0.086
		Log body mass (g)	− 1.49	4.34	− 0.34	0.734
		Levamisole treatment	0.16	0.61	0.26	0.797
Adult	Initial antibody level	Maximum lyre length (cm)	0.17	0.07	5.59	0.012
		Log body mass (g)	− 0.53	1.58	− 0.34	0.739
Adult	Peak response	Maximum lyre length (cm)	0.22	4.99	1.37	0.180
		Log body mass (g)	− 2.84	5.00	− 0.57	0.574
		Levamisole treatment	0.88	0.43	2.05	0.050

### Survival and immune response

**Fig. 3:**
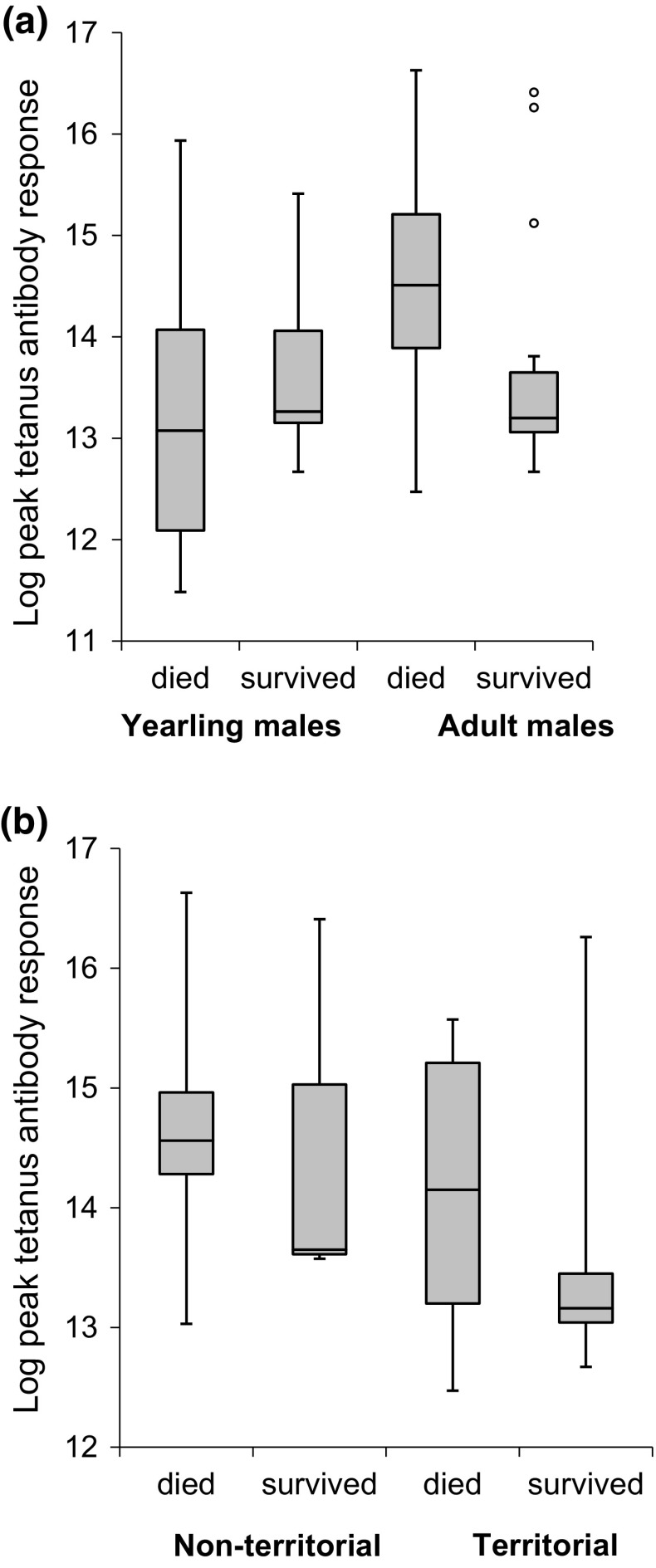
Median ± IQR log peak antibody response level in relation to survival to the following year (died, survived) for **a** adult (*N* = 34) and yearling birds (*N* = 34) and **b** for territorial (*N* = 23 (9 died, 14 survived) and non-territorial (*N* = 11, 8 died, 3 survived) adult birds

As predicted if tetanus was a novel immune challenge, survival was unaffected by baseline antibody levels (*β* ± SE = 0.33 ± 0.34, *Z* = 0.96, *p* = 0.338), age (*β* ± SE = 3.96 ± 7.20, *Z* = 0.55, *p* = 0.582) or its interaction with baseline antibody levels (*β* ± SE = − 0.34 ± 0.56, *Z* = − 0.61, *p* = 0.541). However, survival was significantly related to the interaction between peak antibody response and age (Table 2). Specifically, there was positive selection on antibody response in yearling males, but negative selection in adult males (Fig. 3a). Levamisole treatment did not influence male survival significantly (Table 2).

**Table 2:** Results of GLM models the response of (a) male survival in relation to age (adults and yearlings males combined), peak antibody response, levamisole treatment and the interaction between age and peak antibody response

Response variable	Explanatory variables	*β*	±SE	*z*	*p*
(a) Survival	Peak antibody response	0.49	0.36	1.37	0.132
	Age	17.45	6.97	2.50	0.012
	Levamisole treatment	1.05	0.66	1.59	0.111
	Peak antibody response* Age	− 1.24	0.51	− 2.45	0.014
(b) Mating success	Initial antibody response (linear)	51.05	22.85	2.23	0.026
	Initial antibody response (quadratic)	− 1.94	0.88	− 2.21	0.027
	Levamisole treatment	− 0.48	0.30	− 1.59	0.112
(c) Mating success	Peak antibody response (linear)	18.93	4.59	4.12	< 0.001
	Peak antibody response (quadratic)	− 0.66	0.16	− 4.11	<0.001
	Levamisole treatment	0.32	0.33	0.95	0.344

Models of male mating success were limited to adult males and in relation to linear and quadratic functions of (b) initial antibody response or (c) peak antibody levels and levamisole treatment

**Fig. 4:**
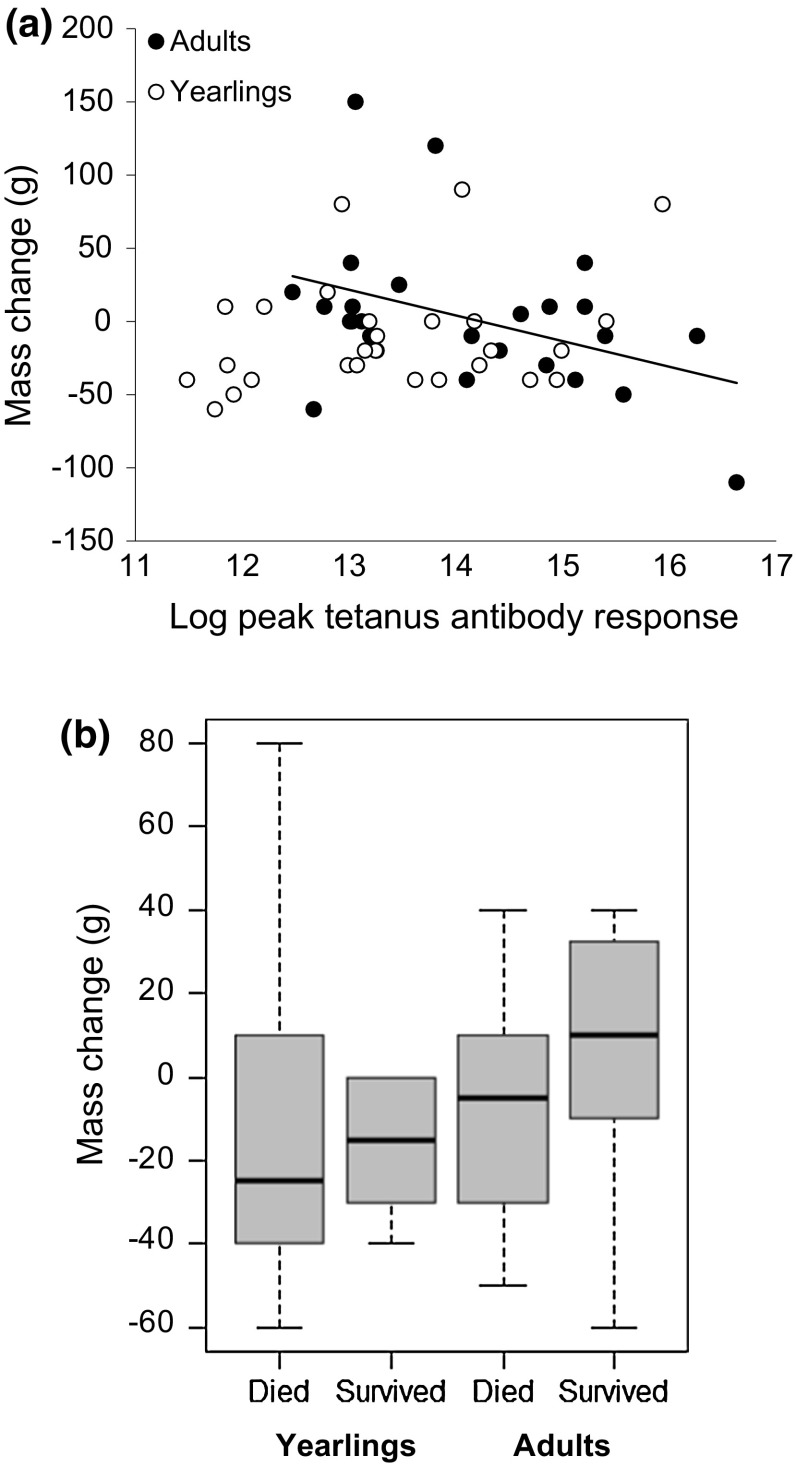
The **a** Change in mass (g) between initial capture and peak immune response in adults (solid points, solid regression line: *y* = 249.83 − 17.55*x*; *N* = 25) and yearlings (open points; *N* = 28) and **b** median ± IQR change in mass (g) of yearling (18 died, 10 survived) and older males (14 died, 11 survived) according to their survival rate to the following year

Mass loss was significantly related to the interaction between male age and peak response (male age x peak response: *β* ± SE = − 26.90 ± 10.15, *t* = − 2.65, *p* = 0.011; male age *β* ± SE = − 385.45 ± 140.34, *t* = − 2.75, *p* = 0.008; peak response: *β* ± SE = − 9.35 ± 7.01, *t* = 1.33, *p* = 0.189). For adults, higher responses were associated with greater mass loss, whereas in yearlings, no such pattern occurred (Fig. 4a). In turn, adults males with greatest mass loss had lower survival (Survival: *β* ± SE = 45.06 ± 19.52, *t* = 2.31, *p* = 0.031; Levamisole: *β* ± SE = − 40.35 ± 19.79, *t* = − 2.04, *p* = 0.054; Fig. 4b), but this was not found in yearlings (Survival: *β* ± SE = 4.76 ± 15.87, *t* = 0.30, *p* = 0.767; Levamisole: *β* ± SE = − 8.23 ± 40.98, *t* = − 0.20, *p* = 0.842).

### Lek attendance, mating success and immune response

There was a tendency for males with higher peak responses to be non-territorial (*β* ± SE = − 0.63 ± 0.36, *z* = − 1.75, *p* = 0.080; Fig. 3b), but there was no effect of levamisole (*β* ± SE = − 0.27 ± 0.80, *z* = − 0.34, *p* = 0.737). Amongst territorial males, there was a tendency for males with higher peak responses to die before the next year (*β* ± SE = − 0.89 ± 0.51, *z* = − 1.74, *p* = 0.082), but there was no effect of levamisole treatment (*β* ± SE = 1.90 ± 1.19, *z* = 1.60, *p* = 0.117).

**Fig. 5:**
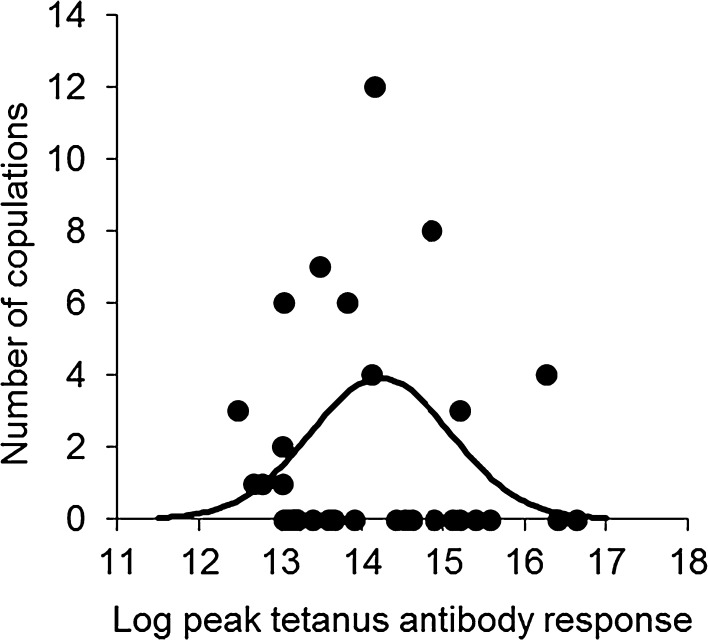
The number of copulations in relation to log tetanus antibody level during the peak response (20–40 days) for adult birds (*N* = 25). The significant quadratic regression line is shown (solid line)

Males with intermediate baseline antibody level had higher male mating success (Table 2), but there was no relationship between levamisole and male mating success (Table 2). Male mating success was significantly related to an intermediate peak response (Table 2; Fig. 5). Again, levamisole treatment had no effect on mating success (Table 2).

## Discussion

### Immune response and tail length

Despite a widespread expectation that sexual ornaments can honestly signal immune responses (Saino et al. 1997; Zuk and Johnsen 1998; Mougeot et al. 2004; Loyau et al. 2005; Bonato et al. 2009), there is considerable inter-species variance in this effect; some studies finding no relationship (e.g. Westneat et al. 2003), others finding that different components of the immune system are reflected in different traits (Bonato et al. 2009). In our study, a long-term measure of male quality (lyre length) was positively related to baseline antibody levels for adult males but not yearlings and tended to be related to peak response in yearlings and adults. Of all traits, lyre length (i.e. tail length) we might be predicted to have a strong relationship to immunocompetence as it is negatively related to parasite load in black grouse (Höglund et al. 1992). Linked to this, tail length has been related to peak antibody response barn swallows *Hirundo rustica* (Saino et al. 1997) and cell-mediated immunity in peacocks *Pavo cristatus* (Møller and Petrie 2002), but not antibodies in pheasants (Smith et al. 2007). A genetic analysis has shown a positive relationship between tail (train) length and MHC diversity in peacocks (Hale et al. 2009), suggesting that males with greater MHC diversity are healthier and, therefore, better able to produce large trains and have better general immunocompetence. Our data confirm the potential for tail length to signal male immunity, but also suggest that such a pattern may also be age-specific. The discordance between adults and yearling may reflect selection against poor quality males in yearlings; as a consequence, only high quality yearling males survive to the following year thereby strengthening the relationship between the sexual signal and baseline antibody response. Alternatively, yearling males may invest more in somatic maintenance. Our results are only indicative of a relationship that tail length may signal some component of immunity, but further work is needed to fully understand the mechanisms and the genes underpinning this.

### Immune response, survival and mating success

Previous studies have shown a positive relationship between immune response and survival in early life stages such as nestlings (Christe et al. 1998; Cichoń and Dubiec 2005). Conversely, there is a negative relationship between a strong immune response and survival in reproductive-aged individuals (Hanssen et al. 2004; Møller and Saino 2004) often mediated by nutrition (Houston et al. 2007; Valtonen et al. 2010). Our results are in line with these earlier studies. Yearling males generally do not invest in lekking activity (Kervinen et al. 2012), and, therefore, individuals might be better able to cope with the high costs of an immune response. In contrast, adults with high responses were less likely to survive than those with weak responses. Lekking is energetically demanding (Vehrencamp et al. 1989), very active males lose a considerable proportion of their body mass (Lebigre et al. 2013) and post-lekking mortality is particularly high (Alatalo et al. 1991). Our results suggest that the costs of mounting an immune response occur in conjunction with the costs of lekking, possibly because of the dual increase in physiological costs of lekking and immunity (Hasselquist and Nilsson 2012; Van Dijk and Matson 2016). We do not have direct evidence of the specific costs of a too-high immune response but these could also include nutrient costs, autoimmunity or oxidative stress (Costantini and Møller 2009; Hasselquist and Nilsson 2012). We showed that adult males had a decline in body mass while mounting their immune response and this was associated with reduced survival; this suggesting that the energetic costs may partly cause the fitness costs we observed in adult males.

Previous studies have also shown that an immune challenge is costly because individuals reduce their reproductive effort (Jacot et al. 2004; Ahtiainen et al. 2005; Rantala et al. 2010; Gershman et al. 2010), but studies have also reported no relationship (Westneat et al. 2003) or even a positive relationship between the level of immune response and reproductive success (Ekblom et al. 2005; Ahtiainen et al. 2006). In black grouse, we found that birds with a higher peak immune response tended to have lower lek attendance and not hold a territory, an important prerequisite of any mating success (Rintamäki et al. 2001; Kervinen et al. 2016), and that males with intermediate peak immune responses had greater reproductive success. In some species, individuals with high reproductive effort have reduced antibody production (Deerenberg et al. 1997; Nordling et al. 1998). Conversely, individuals with too-low immune responses may be poor quality males unable to afford the dual costs of an immune response and lekking (Loyau et al. 2005). Our results suggest that males with highest mating success have reduced immune response, either through a better overall immunocompetence, tolerance of infection or as a result of a trade-off between lekking and antibody production. Either way, these opposite patterns may combine to result in an overall stabilising selection acting on the immune responsiveness.

## Conclusions

In summary, our data indicate that individuals with intermediate immune responses have higher fitness in male black grouse, through the opposite selection acting on survival (dependent on males’ age class) and direct stabilising selection acting on male mating success. Our results demonstrate that different components of fitness may select in different directions on immune response and adds to the small body of work that suggests an intermediate immune response may be optimal (Nordling et al. 1998; Stjernman et al. 2008; Graham et al. 2010). In addition, it reinforces the need to use multiple-components of fitness when examining optimal immunity (Graham et al. 2011). In addition, this study shows how ornaments can honestly signal both baseline and response values in different ways. Future work should integrate these findings into longer-term life history to assess adaptive immune response across and individual’s lifespan and what trade-offs individuals make between different fitness components and immune response.
